# The Immunomodulatory Role of Cell-Free Approaches in SARS-CoV-2-Induced Cytokine Storm—A Powerful Therapeutic Tool for COVID-19 Patients

**DOI:** 10.3390/biomedicines11061736

**Published:** 2023-06-16

**Authors:** Maria Csobonyeiova, Veronika Smolinska, Stefan Harsanyi, Michal Ivantysyn, Martin Klein

**Affiliations:** 1 Institute of Histology and Embryology, Faculty of Medicine, Comenius University, Sasinkova 4, 811 08 Bratislava, Slovakia; 2 Apel, Dunajská 52, 811 08 Bratislava, Slovakia; 3 Regenmed Ltd., Medená 29, 811 08 Bratislava, Slovakia; 4 Institute of Medical Biology, Genetics and Clinical Genetics, Faculty of Medicine, Comenius University, Sasinkova 4, 811 08 Bratislava, Slovakia

**Keywords:** COVID-19, exosomes, cytokine storm, cell-free therapy

## Abstract

Currently, there is still no effective and definitive cure for the coronavirus disease 2019 (COVID-19) caused by the infection of the novel highly contagious severe acute respiratory syndrome virus (SARS-CoV-2), whose sudden outbreak was recorded for the first time in China in late December 2019. Soon after, COVID-19 affected not only the vast majority of China’s population but the whole world and caused a global health public crisis as a new pandemic. It is well known that viral infection can cause acute respiratory distress syndrome (ARDS) and, in severe cases, can even be lethal. Behind the inflammatory process lies the so-called cytokine storm (CS), which activates various inflammatory cytokines that damage numerous organ tissues. Since the first outbreak of SARS-CoV-2, various research groups have been intensively trying to investigate the best treatment options; however, only limited outcomes have been achieved. One of the most promising strategies represents using either stem cells, such as mesenchymal stem cells (MSCs)/induced pluripotent stem cells (iPSCs), or, more recently, using cell-free approaches involving conditioned media (CMs) and their content, such as extracellular vesicles (EVs) (e.g., exosomes or miRNAs) derived from stem cells. As key mediators of intracellular communication, exosomes carry a cocktail of different molecules with anti-inflammatory effects and immunomodulatory capacity. Our comprehensive review outlines the complex inflammatory process responsible for the CS, summarizes the present results of cell-free-based pre-clinical and clinical studies for COVID-19 treatment, and discusses their future perspectives for therapeutic applications.

## 1. Introduction

As of 2023, the coronavirus disease 2019 (COVID-19) pandemic has been successfully contained in most parts of the world thanks to effective vaccination, a high proportion of people having successfully fought it off (with or without vaccination), public health strategies, and effective novel treatment methods. It no longer poses such an immediate global public health and socio-economic threat as it used to, mainly in 2020 and 2021. As the WHO Director-General Tedros Adhanom Ghebreyesus said in March 2023 “I am confident that this year we will be able to say that COVID-19 is over as a public health emergency of international concern” [1]. However, these optimistic future perspectives should be interpreted with caution, because the evolutionary trajectory of SARS-CoV-2 is uncertain. Future local outbreaks and new variants will likely have to be dealt with [2]. Even if future infections are mild from the epidemiological perspective and reserved for local epidemics, COVID-19 will not cease to be a dangerous disease with many potentially life-threatening complications resulting from the dysregulated release of proinflammatory cytokines, known as the cytokine storm (CS) [3]. These are only some of the scenarios to consider and prepare for to diminish the debilitating effect of a future pandemic [4]. Pointing toward possible future complications are issues such as the emergence of new variants and the global disparities in vaccine distribution and access. Recently, Wang et al. reported alarming antibody evasion properties in Omicron subvariants BQ and XBB, which, due to the mild clinical presentation of Omicron infection, do not pose an urgent problem; however, this tendency to evade antibodies could significantly hinder vaccination strategies [5].

Moreover, there is still the relevant issue of long-COVID, which remains a persistent problem for many patients around the globe, with the cumulative prevalence ranging between 9 and 63% [6]. Therefore, the development of state-of-the-art therapeutic approaches combating COVID-19 concerning CS-related complications is still a timely area of research, which can provide better outcomes for COVID-19 patients but also can serve as a foundation for the development of therapeutic strategies for potential pandemics that could strike in the future. Many approaches have been implemented to combat CS-induced COVID-19-associated complications, e.g., corticosteroids, hydroxychloroquine, chloroquine, or tocilizumab [7]. There are also strategies that attempt to implement knowledge regarding the immunoregulatory effect of mesenchymal stem cells (MSCs) and their derivatives (conditioned media (CMs), or more specifically, exosomes forming the base of cell-free therapies). Among different cell populations, MSCs undoubtedly possess numerous benefits as a cell-based treatment for COVID-19 patients, as evidenced by several already-finished and countless ongoing clinical studies [8]. According to the specific abilities of MSCs, such as the regulation of innate and adaptive immune response, they are indisputable candidates not only for COVID-19 but also for other immune-mediated inflammatory diseases. However, MSCs may present limitations in delivery, safety, proper engraftment, differentiation, and overall therapeutic efficacy.

On the other hand, MSC derivatives, such as CMs or extracellular vesicles (EVs), are more clinically suitable thanks to their fundamental properties in immunomodulation and wound healing [9,10]. Moreover, according to extensive research, it is now widely accepted that MSCs are therapeutically effective mainly due to their paracrine secretion that mediates cell-to-cell signaling. On account of this, it was proved by several studies that MSC derivatives possess therapeutic effects similar to MSCs themselves, involving anti-inflammatory, regenerative, proangiogenic, and anti-protease qualities [11,12]. Regarding the transport of MSCs and their derivatives, MSCs are usually transferred to the body intravenously. On the contrary, thanks to the small size of EVs, there might be an alternative route of administration, such as inhalation, enabling direct pulmonary delivery [13]. Therefore, MSC derivatives can be more convenient and safer, with more significant potential for clinical translation. Other benefits of MSC derivatives include eliminating host immune reactions; the possibility of long-term storage without harmful content in cryopreservation media and without significant loss in bioactivity; and overall cost-effectivity, enabling their mass production [14].

This review paper aims to provide a comprehensive overview of cell-free approaches to SARS-CoV-2-induced CS as a tool for the state-of-the-art COVID-19 therapeutic strategy.

## 2. Immunopathogenesis of COVID-19

Immunopathogenesis is generally defined as a mode of disease development with a substantial contribution of innate and adaptive immune system mechanisms, which, upon dysregulation, cause collateral damage, promoting further disease development and its complications [15]. In 2005, Dandekar and Perlman published a paper on SARS-CoV, a virus responsible for SARS infection wreaking havoc between 2002 and 2004. The authors reported that a dysregulated immune response is essential to its pathogenesis, which can lead to life-threatening complications. Ironically, the authors concluded that “lessons from such studies will help us to understand more about the pathogenesis of SARS in humans and to prevent or control outbreaks of SARS in the future” [16]. Less than two decades later, a new SARS-CoV-2 strain struck the world, bringing severe socio-economic and public health challenges. Not only had this new outbreak failed to be prevented, but it was also more severe by several orders of magnitude than the original SARS-CoV virus. Fortunately, many insights, research endeavors, and experiences from fighting the original virus helped us understand the foe better. From the immunopathogenic perspective, the most dreaded complication during COVID-19 is immune system dysregulation, known as CS. Alternatively, it can be referred to as cytokine release syndrome or cytokine-associated toxicity. It is characterized by hyperinflammation, which is damaging to the surrounding tissues. Its hallmark is excessive cytokine release which fails to contain the immune system response to the harmful pathogen elimination and orchestrate the resolution of inflammatory responses to the previous baseline state [17]. In the case of inadequate treatment, it can lead to multiorgan dysfunction or even multiorgan failure with possibly fatal consequences [18]. Initially, COVID-19 was considered a respiratory condition, primarily affecting the lungs and causing viral pneumonia. Soon after, a thorough investigation of the SARS-CoV-2 and COVID-19 pathogenesis revealed that altered immune system regulation is integral to COVID-19 multiorgan involvement and severe extrapulmonary complications. The first indication that COVID-19 should be considered from the immunopathogenic standpoint was a change in patients’ leucogram, which showed lymphopenia and an enhanced neutrophil–lymphocyte ratio [19,20]. Fighting off the SARS-CoV-2 virions and subsequent COVID-19 development is heavily influenced by the components of the innate and adaptive immune systems alike.

Innate immune responses are mediated through the interaction between viral pathogen-associated molecular patterns (PAMPs) and pattern recognition receptors (PRRs), e.g., Toll-like receptors (TLRs) on the surface of host innate immune cells, such as neutrophilic granulocytes, macrophages, and NK cells, leading to the secretion of proinflammatory cytokines. The adaptive immune response is mediated by the recruitment of CD8+ cytotoxic T-lymphocytes (CTLs), CD4+ helper T-lymphocytes (Th cells), and antibodies-producing B-lymphocytes [21], although B-lymphocytes usually do not contribute to the CS [18]. CD4+ helper T-lymphocytes are differentiated into subclasses, namely Th1, Th2, Th9, and Th17. Th1 and CTLs are routinely identified as cells fighting off viral infections, whereas the excessive Th1 response is most often associated with CSs [18]. Especially significant is the population of CD4+ PoxP3+ regulatory T-lymphocytes (Tregs), whose primary role is the attenuation of immune responses and keeping them adequately reserved to the noxious stimuli without overactivation, which is deleterious to the host tissues. They can be thought of as cells responsible for immune homeostasis. In the case of COVID-19, the proper function of Tregs protects from hyperinflammation and CS-induced tissue damage. Therefore, Treg targeting has been used as a promising therapeutic approach [22]. A delicate balance of all these cell populations is essential for the immune reaction to kill off the invading pathogens with minor damage to healthy surrounding tissues. Unfortunately, the exact opposite occurs during COVID-19.

An important immunopathogenic factor in COVID-19 is immune cell exhaustion, broadly defined as immune cell fatigue resulting from a robust initial immune response [23]. Xang et al. reported that CS might promote T-lymphocyte exhaustion [24]. This combination of immune cell dysfunction and hyperinflammatory CS leads to a poor COVID-19 prognosis. The panel of cytokines responsible for the CS is vast. As reviewed by Montazersaheb et al., cytokines involved in the COVID-19 immunopathogenesis include the Interleukin (IL)-1 family, IL-6, IL-10, IL-17, interferon-gamma (INF γ)-inducible protein 10 (IP-10), and tumor necrosis factor-alpha (TNF-α) (a more proper official term, TNF, will be used from now on [25]), among many others [26].

The IL-1 family of cytokines comprises IL-1, IL-18, IL-33, IL-36, IL-37, and IL-38. Collectively, these proinflammatory cytokines activate NLRP3 inflammasomes [27], leading to cell death VIA pyroptosis [28]. On the one hand, this type of cell death has been associated with host resistance to pathogen invasion. However, on the other hand, pyroptosis can bring about overwhelming hyperinflammation [29].

IL-6 is the cardinal cytokine implicated in the immunopathogenesis of CS, also serving as a predictor of COVID-19 clinical outcomes and mortality [30]. In 2020, Coomes and Haghbayan published a systematic review and meta-analysis which revealed that COVID-19 patients with a complicated course of the disease have a 2.9-fold increase in serum IL-6 concentration compared to those with mild symptoms and good outcomes. IL-6 levels correlated with adverse clinical outcomes such as ICU admission, ARDS development, or death [31]. Considering the crucial role of IL-6 in COVID-19 prognosis, many studies have targeted IL-6 pathways as a potential therapeutic approach. One of the many drugs tested for COVID-19 management is tocilizumab—an IL-6 receptor (IL-6R) inhibitor. Galván-Román et al. retrospectively studied COVID-19 inpatients and found that IL-6 levels above 30 pg/mL best predicted the need for invasive mechanical ventilation. The administration of tocilizumab improved oxygenation and decreased mortality [32].

IL-10 is another cytokine which is significantly elevated during COVID-19 and correlates with poor clinical outcomes. Although generally functioning as an anti-inflammatory cytokine, its high levels in severe COVID-19 may seem counterintuitive at first glance. There are several hypotheses on the possible mechanisms behind its action. Islam et al. reported that IL-10 fails to suppress the hyperinflammatory state, or its anti-inflammatory activity is not universal [33]. The pleiotropic activity of IL-10 was also discussed by Lu et al., who hypothesized that IL-10 could act as a proinflammatory cytokine under certain conditions [34]. An alternative explanation has similar features as the first one. It is the state of “IL-10 resistance”, defined as the inability of immune cells to respond to IL-10-mediated immune suppression appropriately. This explanation is corroborated by the observations of severe COVID-19 in patients with type II diabetes, who were previously shown to have diminished IL-10 action due to hyperglycemia [33,35]. The meta-analysis and regression published by Dhar et al. showed that out of the many cytokines elevated in COVID-19, IL-10 and IL-6 alone are sufficient to determine and predict the risk of the severe clinical course of the disease, allowing for early interventions which will result in better prognoses [36]. The important role of IL-6 and IL-10 is rooted in their ability to regulate antibody production by influencing the differentiation and maturation of B-lymphocytes [37]. Heine et al. conducted a clinical trial where they found that autocrine IL-10 secretion by B-lymphocytes themselves induced their differentiation into IgM- or IgG-secreting plasmablasts [38]. The effective production of protective antibodies is generally associated with a good COVID-19 outcome; however, there have been reports on harmful autoantibodies whose production can be life-threatening. Bastard et al. found that severe COVID-19 patients can produce IgG antibodies against type I interferons (IFNs) which normally help block SARS-CoV-2 infection [39]. The detrimental role of IL-6 and IL-10 was previously reported in various autoimmune disorders [40,41].

Type III IFNs are anti-viral cytokines comprising four IFN-λs which are vital for the normal regulation of anti-viral response in the respiratory system. Their central role is to limit the infection by the means of viral resistance induction. IFN-λs are anti-inflammatory and responsible for tissue protection. In the context of COVID-19, IFN-λs can be regarded as CS preventers [42]. Moreover, Broadbent et al. found that the endogenously activated IFN-λ1 pathway provides resistance against SARS-CoV-2 infection [43].

IL-17 (IL-17A) is the proinflammatory cytokine produced by Th17 cells. Of the many cytokines produced upon infection with SARS-CoV-2, IL-17 is most often associated with pulmonary inflammation. Along with IL-6 and IL-8, IL-17 seems to be a principal cause of pulmonary fibrosis. IL-17 is also a potent activator of other proinflammatory cytokines released from various lung alveoli cells, including epithelial cells, endothelial cells, and alveolar macrophages [44]. Maione et al. summarized that IL-17 could be associated with COVID-19 severity and progression. They described it as a “rheostat” of the COVID-19 immune response [45].

IP-10 is a chemokine synthesized by various cells as a response to INFγ. IP-10 regulates CD4+ and CD8+ T cells, natural killer cells, and dendritic cells. Bunprakob et al. studied plasma samples of patients with various disease severity and concluded that IP-10 elevation is directly proportional to disease severity [46]. Chen et al. also reported that serum IP-10 could be a biomarker of COVID-19 severity and is associated with mortality risk [47]. Similar results were published by Mulla et al., who evaluated a panel of four cytokines, including IP-10, and found that its serum levels are significantly higher in critically ill patients [48].

TNF is a pleiotropic cytokine involved in regulating acute and chronic inflammation. It can mediate apoptosis, cell proliferation, and the release of other cytokines. Physiologically, it controls tumor formation and immune system homeostasis. Its dysregulation can lead to CS in COVID-19 patients [49]. Jia et al. reported serum TNF as an independent risk factor for death in patients with a severe course of the disease [50]. Some authors view TNF as the central mediator of CS, implicating it in blood clotting activation, lung damage, or even heart failure. Thus, the therapeutic targeting of NF-κB-mediated TNF signaling might significantly decrease mortality rates [51].

## 3. Immunomodulating Properties of Conditioned Media (CM)

CM is a secretion rich in multiple critical factors, such as cytokines, chemokines, growth factors, several soluble proteins, enzymes, bioactive lipids, EVs, microvesicles, and exosomes produced by MSCs during in vitro cultivation [52,53]. The main therapeutic factors found in CM, which protect alveolar epithelium and endothelium against injury, are keratinocyte-growth factor (KGF) and angiopoietin-1. On the other hand, the anti-inflammatory properties possess IL-10, IL-13, IL-1, IL-18 binding protein, ciliary neurotrophic factor (CNTF), neurotrophin-3 (NT-3) factor, prostaglandin-E_2_ (PGE_2_), TNF-stimulated gene-6 (TSG-6), TNF-β1, and Lipoxine A_4_ [54,55,56,57]. CM also obtain a couple of anti-microbial factors, such as Lipocalin-2 and peptide LL-37 [58], and an anti-fibrotic factor—milk fat globule EGF factor 8 (MFGE8) [59]. It is essential to mention that MSCs also release proinflammatory cytokines (IL-1β, IL-6, IL-8, and IL-9), so the variable composition of a secretome is greatly influenced by the physiological state of MSCs, pathological conditions (hypoxic cultures), 3D culture conditions, and other distinct extrinsic factors [60,61]. Generally, CM can be prepared using a protocol involving four steps: the isolation and characterization of cells, cultivation in the proper cultivation medium, cell proliferation, and finally, CM collection with subsequent ultrafiltration [62].

Several improvements are being applied to enhance CM’s therapeutic effect, including the exposure of cells to hypoxic conditions, hyperoxic pre-conditioning, pre-conditioning with growth factors or hormones, and 3D cultivation where the cells are grown as spheroids [63,64,65,66]. In particular, it was found that stem cells cultivated under hypoxic pre-conditions (1% O_2_ for 24 h) significantly increased the secretion of TNF-α, HGF, bFGF, VEGF, IL-10, IL-6, and IL-8 [67,68,69]. Moreover, according to a study by Chen et al., the CM fraction obtained from hypoxic MSCs enhanced the proliferation of specific cells, such as keratinocytes, fibroblasts, and endothelial cells, thus contributing to skin wound healing. In another study published by Yu et al., the hypoxic pre-conditioning of MSCs enhanced the efficacy of MSC-CM in the anti-inflammatory polarization of microglia by inhibiting the expression of proinflammatory cytokines, such as IL-1β, IL-6, CD86, and inducible nitric oxide synthase, while, in contrast, upregulating IL-10. On the other hand, the hyperoxic pre-conditioning of MSCs enhanced the secretion of the bioactive anti-inflammatory molecule—intestinal secretin tumor cell line (STC-1)—which is a potent antioxidant with the ability to increase the resistance of cells to damage caused by hypoxia [70]. Additionally, several authors reported the favorable properties of CM secreted from spheroids [65,71,72]. For example, Ylöstalo et al. cultured MSCs as 3D spheroids and reported enhanced the anti-inflammatory properties of the spheroids’ CM in terms of the inhibition of proinflammatory cytokines’ (TNF-α, CXCL2, IL-6, IL-23, and IL-12p40) secretion by macrophages, and on the other hand, the increased secretion of anti-inflammatory cytokines (IL-10 and IL-1a). The authors suggested that the immunomodulatory activity of spheroid CM is responsible for elevated levels of PGE_2_ which can change the proinflammatory M1 macrophage phenotype to the anti-inflammatory M2 phenotype [65]. Advanced approaches also include the genetic modification of MSCs to increase the secretion of some specific factors into the CM. Genetic manipulation techniques, such as CRISPR/Cas9, can boost the production of factors involved in stemness, aging, proliferation, or immune response [73].

Until today, numerous studies published positive outcomes of MCS-CM on the improvement of several pathological processes present in acute lung injury (ALI); inflammatory lung disease; skin wounds; periodontitis; neurodegenerative diseases, such as Parkinson’s and Alzheimer’s disease; or other various immune-mediated inflammatory diseases [62,74,75,76,77,78,79,80,81,82,83]. For instance, in studies published by Goolaerts et al., Ionescu et al., Su et al., Ding et al., Zhou et al., and Tang et al., the authors tested the effect of MSC-CM in vitro and in vivo in rodent models of ALI. It is well known that the typical pathophysiological features of ALI are lung inflammation, changes in alveolar ion transport, and increased endothelial permeability, often resulting in acute respiratory distress syndrome (ARDS). In all the mentioned research, the administration of MSC-CM resulted in a reduction in lung tissue inflammation and enhancement in wound healing, mainly through activating alveolar macrophage (AM) function; the reduced expression of anti-apoptotic molecules accompanied by increased neutrophil apoptosis; the increased expression of miR-214 and miR-34c; and the downregulation of proinflammatory cytokines, such as IL-1β, IL-6, and TNF-α [62,75,77,84,85,86]. A similar affirmative result was published by Su et al., who found out that CM obtained from induced pluripotent stem cells (iPSCs) can attenuate endotoxin-induced ALI on mice model via the significant enhancement of endogenous leukemia inhibitory factor production and a decrease in the transendothelial migration of neutrophils [87]. In a study published by Li et al., the authors observed improved ventilator-induced lung injury (VILI) and the restoration of the bronchial structure after treatment with iPSC-CM. In this case, the CM suppressed the production of macrophage inflammatory protein-2 (MAP-2) and malondialdehyde. An increased level of interferon gamma-induced protein 10 (IP-10) was also detected [88]. According to Schnabel et al., iPSCs have comparable immunogenic and even more potent immunomodulatory properties compared to bone-marrow-derived MSCs (BM-MSCs) or umbilical-cord-derived MSCs (UC-MSCs); therefore, the use of CM obtained from iPSCs could represent a promising alternative to BM-MSC-CM [89].

All in all, the studies above proved that the therapeutic potency of CM obtained from stem cells is similar to stem-cell-based therapies (Table 1). However, some limitations also exist, hampering the use of CM, such as a lack of standard cultivation protocols and an insufficient understanding of CM potency, which affects the application of the proper therapeutic dose, volume, and route of administration. Overall, the safety and potency of CM are still restricted by the absence of information about the complete multifactorial characterization of all CM components [13].

## 4. Immunomodulating Properties of Extracellular Vesicles

EVs are heterogenous cell products characterized as nano-sized vesicles encapsulated by a lipid bilayer. EVs contain various compounds involved in immune regulation, inflammation activation, and inflammation-related complications. As well as MSC-CM, EVs derived from MSCs (MSC-EVs) are extensively studied as promising therapeutical tools for various diseases [90]. Based on the size and biogenesis of EVs, they are divided into three subtypes: exosomes (30–150 nm), microvesicles (0.1–1 µm), and apoptotic bodies (1–5 µm). They can also be divided according to their origin into exosomes—microvesicles, microparticles, or large vesicles and endosomes, which are of endosomal origin representing exosomes [91,92,93]. The classification of various EVs is still not uniform due to researchers’ lack of consensus on accurate surface markers. According to the International Society for Extracellular Vesicles (ISEV), EVs should be categorized according to their size, density, and biological makeup [94]. In general, there are several methods used for EVs’ isolation, including the gold-standard differential and gradient density centrifugation/ultracentrifugation, filtration, immune-affinity isolation, size-exclusion chromatography, polymeric precipitation, and microfluidics-based techniques [95]. Among the mentioned methods, microfluidics-based technologies provide the highest levels of purity and recovery for exosome isolation, detection, and analyses compared to others [96].

Among EVs, exosomes are the most potent ones from a regenerative medicine perspective, thanks to their cargo’s specific content, which is responsible for their anti-apoptotic, anti-bacterial, immunomodulatory, and anti-fibrotic properties. Moreover, they play a crucial role in intercellular communication, altering the recipient cells’ fate. Within the exosomes’ lipid membrane is enclosed a complex cocktail of bioactive molecules, including nucleic acid, microRNA (miRNAs), lipids, proteins, and even metabolites [97,98,99]. Crucial exosomal components are specific miRNAs that participate in intercellular communication, affect the phenotype and development of immune cells, mediate microenvironmental changes, and regulate the pathological processes in various diseases, including lung diseases [100]. These highly conserved single-stranded small non-coding RNA molecules are extremely stable and are resistant to low acidic pH and RNase-mediated degradation [101]. It was shown that several miRNAs, such as miR-126, miR-150, miR-451, miR-30b-3p, miR-132-3p, miR-146a-5p, and miR-182-5p, can ameliorate lung damage in ALI/ARDS, reverse the progression of lung disease, and have a protective effect by regulating macrophage function [102,103,104,105,106,107,108].

It is essential to mention that the effects of exosomes on the different target cells can differ from pro-survival to immunomodulatory to pro-apoptotic due to their functional heterogeneity. Behind the immune regulation of exosomes lies the induction of several signaling pathways via exosomes’ surface ligands, the transfer and direct or indirect presentation of antigenic peptides, and the delivery of DNA-inducing cGAS-STING signaling in recipient cells [109]. Exosomes possess several advantages over stem-cell-based therapies, including the presence of a lipid membrane in which essential therapeutic molecules are encapsulated, so they are protected from degradation; secondly, the durability of exosomes and easy handling manufacturing; and lastly, the more accessible storage possibilities thanks to their small size [92]. In addition, their use is minimally associated with immune rejection or tumor-formation issues because they cannot proliferate or differentiate inside the recipient’s body. Moreover, recent exosome engineering techniques allow the delivery of various therapeutic molecules, such as chemotherapeutic agents, short-interfering RNAs, immune modulators, or antisense oligonucleotides. Via the targeted delivery of exosomes, it is possible to increase the therapeutic concentration and minimize potential side effects [93,110]. Due to their unique ability to interact with recipient cells, the use of exosomes is preferable over the use of CM, and in recent years, they have been increasingly applied in the treatment of several diseases [60]. For instance, therapy using exosomes resulted in positive outcomes in numerous conditions such as respiratory disorders [111,112], urinary tract infections [113], intestinal infections [114,115], wound infections [116,117,118], neurodegenerative diseases [118,119,120,121], liver diseases [122,123,124], cardiovascular diseases [125,126,127], and sepsis [128,129].

Among respiratory diseases, the effects of exosomal therapy have been the most extensively studied in ALI and related ARDS [112,130,131,132,133]. According to Shah et al., BM-MSC-derived exosomes play a critical role in the recovery process for patients with ARDS thanks to the expression of Runx1 isoforms (Runx1p66/p52), which help to facilitate endothelial cell proliferation and recovery [134]. Other authors also reported significant benefits of MSC-derived exosomes/microvesicles on ALI/ARDS regarding inflammation reduction and the regeneration of the alveolar epithelium. Both Zhu et al. and Monsel et al. tested the effects of MSC-microvesicles’ administration in a mouse model of bacterial-pneumonia-induced ALI and found that microvesicles improved survival in part through KGF secretion and decreased the influx of inflammatory cells, cytokines, and bacteria in injured lung tissue [111,135]. According to Tang et al., their partial role in overall improvement of inflammation in ALI may also be attributed to the overexpression of angiopoietin-1 mRNA [130]. For the first time, Khatri et al. demonstrated the therapeutic effect of MSC-EVs in the pre-clinical pig model of ALI caused by the influenza virus. Intratracheally administrated MSC-EVs successfully inhibited virus replication in the lungs, virus-induced apoptosis, and the production of proinflammatory cytokines [136]. More recently, Xia et al. reported a critical function of transferring mitochondria components via exosomes from MSCs into the target alveolar macrophages in an ALI mouse model, whereas these mitochondrial components were able to mitigate lung inflammation by shifting macrophages to the anti-inflammatory phenotype [137].

Based on extensive research evidence on the therapeutic effect of exosomes in ALI/ARDS (Table 2), their clinical application in COVID-19 is right next door.

## 5. The Role of CMs in Cell-Free Therapy of COVID-19

Recently, pre-clinical studies and articles reported on the comparable therapeutic effect of CM on the application of stem cells. This led us to study the importance of CM in cell-free therapy approaches for treating various diseases, e.g., COVID-19. Cell-free therapy involves utilizing the therapeutic effects of bioactive molecules secreted by cells rather than using the cells themselves. The effects of MSC products are introduced in Table 3. They can act in many ways, ranging from mediators through regenerative agents to important immunomodulatory substances [138]. More than 10 years ago, Fang et al. demonstrated that angiopoietin-1 secretion was responsible for beneficial effects on the restoration of epithelial protein permeability in a culture of human alveolar type II cells [139]. Additionally, MSC-derived EVs are studied in association with the treatment of acute respiratory distress syndrome (ARDS) because they can carry various molecules (e.g., transcription factors, RNAs, lipids, …) and even organelles such as mitochondria [140].

In COVID-19, CM derived from specific cell types can be harnessed to modulate the immune response, promote tissue regeneration, and potentially inhibit viral replication [141]. It was demonstrated that CM from specific cell types, such as MSCs, have been shown to possess potent anti-inflammatory properties. These media can contain soluble factors that dampen excessive immune responses, reduce inflammation, and prevent CSs associated with severe COVID-19 [142]. However, CM pose a problem in standardizing the methodology of production and validation, as changes in these factors were found to produce different results [143].

Several pilot studies using MSC therapy to overcome COVID-19 symptoms provided promising results. For instance, Bari et al. proposed cell-free (MSC secretome) therapy to treat COVID-19 fibrotic lungs through its anti-inflammatory and anti-fibrotic factors [144]. Pati et al. demonstrated a positive effect of MSC-based CM on the restoration of the permeability of the pulmonary endothelia, both in vitro and in vivo. Moreover, they showed that MSC-CM can inhibit the systemic levels of inflammatory cytokines and chemokines in the serum of treated rats [145]. In another study, Goolaerts et al. found that MSC-based CM reversed epithelial hyperpermeability and epithelial Na+ transport, thus preventing the development of acute lung injury [75].

Lapuente and colleagues showed that the co-culture of adipose-tissue-derived MSCs and macrophages M2 led to a significant increase in IL-6 and typical anti-inflammatory cytokine, IL-1Ra, and tissue inhibitor of metalloproteinases (TIMP), TIMP-1. They recorded increased concentrations of regenerative growth factors HGF and IGF-1 and matrix metalloproteinases (MMPs) MMP1 and MMP-3. On the other hand, in the case of cytokines involved in inflammatory processes (e.g., MCP-1, PDGF-BB, and VEGF-A), they observed a significant decrease. In addition, they showed that the CM was not toxic on human PBMC or THP-1 monocytes and prevented lipopolysaccharide (LPS)-induced growth effects on those cell types [146].

High mobility group box 1 (HMGB1) was found to be crucial for SARS-CoV-2 susceptibility regulation. Gowda et al. studied the effect of Glycyrrhizin (HMGB1 inhibitor) on macrophages cultured in CM from lung cells expressing SARS-CoV-2 S-RBD and Orf3a. Glycyrrhizin lowered virus replication and the release of ferritin and proinflammatory cytokines (IL-1β, IL-6, and IL-8) [147].

Bhardwaj et al. explored a novel approach for SARS-CoV-2 treatment and prevention. They used CM and a modified exercise cell culture to generate myokines by differentiating the C2C12 cells to myotubules and inducing them into contractions. CM was later applied to the immortalized human bronchial epithelium cell line combined with CM from unstimulated cells. The comparison of CM-stimulated and unstimulated cells led to the discovery of a 32% reduction in the expression of ACE2 and 41% of TMPRSS2 mRNA, showing a possible reduction in SARS-CoV-2 susceptibility, also usable in similar viruses [148].

Research based on the observed decreased severity of SARS-CoV-2 infections in patients with cystic fibrosis (CF) led Kummarapurugu et al. to study the relationship between high levels of neutrophil elastase (NE) and epithelial ACE-2. CM was used to expose primary human bronchial epithelial (HBE) cells to NE or a control vehicle. NE-treated HBE cells exhibited decreased spike protein binding to HBE and the release of ACE-2 ectodomain fragments, resulting in decreased susceptibility to SARS-CoV-2 infection [149].

A purified spike (S) protein of SARS-CoV-2 and its mediation of virus entry into the host cell was studied by Barhoumi et al., where they described cytokine response, inflammatory markers, oxidative stress, and THP-1-like macrophage polarization in peripheral mononuclear cells, macrophages, and human umbilical vein endothelial cells (HUVECs). In this study, CM were used to treat the HUVEC cells with THP-1 macrophages stimulated by the S protein, which resulted in induced apoptosis and MCP-1 expression, reversible via the use of angiotensin-converting enzyme inhibitors (ACE-I), in this case, perindopril [150]. The S protein was targeted by Kuate et al., who aimed to develop exosomal vaccines by inserting the S protein into MSC-derived EVs. CM were used to treat 293T cells to produce S-pseudotyped vectors, incorporate the proteins into vesicles, and harvest the created EVs containing anti-S1 antibodies [151]. Chaturvedi et al. adopted a different approach to COVID-19. They used SARS-CoV-2 therapy in which the intranasal prophylactic and therapeutic administration of lipid-nanoparticle therapeutic interfering particles (TIPs) suppressed the virus efficiently, reduced proinflammatory response, and prevented pulmonary edema. TIP-treated cells were cultivated in Matrigel in the presence of a human airway organoid (HAO) medium [152].

Finally, yet importantly, IFN-λ-containing CM should be implemented in the future considering the abovementioned crucial role of IFN-λs (type III IFNs) in anti-viral action and protection against the CS. Its profound therapeutic effect was demonstrated in a randomized controlled adaptive platform trial published by Reis et al. who studied the efficacy of a single dose of pegylated IFN-λ. The main finding was that the experimental group had significantly lower hospitalization rates and emergency room visits [153]. Similarly, Santer et al. highlighted the effect of IFN-λ on accelerated SARS-CoV-2 clearance [154]. IFN-λ CM can be manufactured by transfecting the IFN-containing plasmid, as reported by Guo et al. [155].

This shows the vast potential of CMs for their anti-inflammatory, immunomodulatory, regenerative, and anti-viral properties and for modeling conditions to simulate cell responses.

## 6. The Role of Exosomes in Cell-Free Therapy of COVID-19

As mentioned above, exosomes have gained significant attention as potential therapeutic agents for various diseases, including COVID-19. In COVID-19, exosomes have shown promise in cell-free therapy due to their unique properties [91,156]. MSC-derived exosomes provide various routes of action for COVID-19 therapy.

Exosomes help modulate the immune response in COVID-19, during which, the immune response can become dysregulated, leading to excessive inflammation and tissue damage. MSC-derived exosomes suppress proinflammatory cytokines and promote the production of anti-inflammatory factors, thereby potentially reducing the severity of the disease [157,158].

Moreover, exosomes can carry anti-viral molecules, such as microRNAs, which can inhibit viral replication and propagation. Researchers have explored the potential of using exosomes loaded with anti-viral molecules to target SARS-CoV-2, the virus responsible for COVID-19. These exosomes can potentially interfere with viral entry, replication, or the host’s immune response to the virus [159,160].

**Figure 1:**
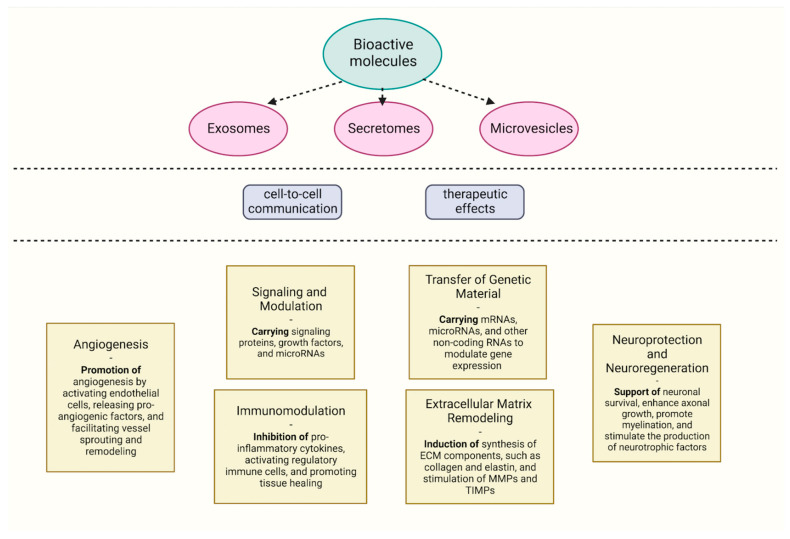
Mechanisms of action of microvesicle-, exosome-, and secretome-derived bioactive molecules in cell-free therapeutic approaches.

It is well known that COVID-19 can lead to significant damage to various organs, particularly the lungs. Exosomes derived from stem cells or specific cell types possess regenerative properties and can promote tissue repair. These exosomes can deliver bioactive molecules to damaged tissues, stimulate cell proliferation, reduce fibrosis, and support the regeneration of injured cells [161]. Figure 1 summarizes the various mechanisms of action of bioactive molecules.

Lastly, MSC-derived exosomes can serve as natural nanocarriers for therapeutics due to their ability to encapsulate and protect various molecules, including drugs. This property has been utilized to deliver anti-viral drugs, anti-inflammatory agents, or other therapeutic molecules directly to the affected tissues. Exosomes can enhance drug stability, bioavailability, and targeted delivery, potentially improving their efficacy and reducing side effects [162,163].

Sengupta et al. performed a non-randomized open-label cohort trial on the safety and effectiveness of exosomes (ExoFloTM) produced from allogeneic bone marrow MSCs for treating severe COVID-19. The goals were to assess safety, including infusion reactions and any adverse events; as well as efficacy, which included overall status as evidenced by disposition; oxygenation as evidenced by the partial pressure of arterial oxygen to fraction of inspired oxygen ratio (PaO_2_/FiO_2_) and oxygen support requirements; degree of inflammation; and immunocompetence as evidenced by levels of C-reactive protein (CRP), D-dimer, and ferritin [164].

This prospective open-label experiment on COVID-19 therapy indicated that the bone-marrow-derived product, ExoFlo, may be safely provided via intravenous infusion. In conclusion, ExoFlo is a viable therapy option for severe COVID-19 due to its safety profile, ability to restore oxygenation, downregulate CS, and reconstitute immunity. ExoFlo’s therapeutic potential must be determined in future randomized controlled trials [164].

Another work published by Prof. Loh’s lab indicates that the terminal complement activation complex C5b-9 can directly trigger the neutrophil release of neutrophil extracellular traps (NETs) and IL-17 [165]. Through a CD59-dependent mechanism, MSC exosomes can decrease complement-mediated neutrophil activation. Exosomes were isolated from immortalized E1-MYC 16.3 human embryonic stem cells. The study demonstrated a feed-forward loop between two key immunologic indicators of severe COVID-19: complements and neutrophils. It also provided an overarching mechanistic context for the association of elevated complement activation, neutrophils, NETosis, and IL-17 with poor COVID-19 outcomes. This work further established that MSC exosomes may selectively block this complement/neutrophil axis via CD59 expression, providing a scientific foundation for using MSC exosomes in treating critically sick COVID-19 patients. The findings presented also made a convincing case for using MSC exosomes to treat severe COVID-19 [165].

It has been demonstrated that exosomes, derived from bone marrow MSCs that underwent an expansion and were encouraged to develop into neurotrophic and immunomodulatory factors secreting MSCs (Exo MSC-NTF), are a promising and innovative biological therapy for ARDS [166]. The pre-clinical study conducted by Kaspi et al. provided a comparison of the efficacy of exosomes isolated from naive MSCs (Exo MSC) and Exo MSC-NTF based on the ability to treat ARDS in a lung damage lipopolysaccharide (LPS) mouse model. The expression of four proteins was evaluated, of which, LIF and AREG were found to be significantly increased in Exo MSC-NTF compared to Exo MSCs. Exo MSC-NTF administration enhanced lung histology and function, raised blood oxygen saturation, and decreased inflammatory cytokines and coagulopathy biomarkers. Exo MSCs also improved, albeit at a slower pace than Exo MSC-NTF. As a result, improved outcomes in mice treated with Exo MSC-NTF may be due, at least in part, to greater lung delivery of factors such as LIF and AREG. However, other variables may contribute to Exo MSC-NTF’s higher positive impact [166].

It has been established that exosomes can enter the bronchioles and alveoli immediately after nebulization, allowing for the highest medicine uptake [167].

Chu et al. conducted a preliminary study of nebulization treatment using exosomes from umbilical cord MSCs on seven patients with COVID-19 pneumonia. Numerous ultrafiltration stages were used to extract and purify exosomes released by MSCs. Every patient received MSC-derived exosomes through nebulization, and the primary safety and effectiveness results were assessed. There were no adverse effects recorded during the safety evaluation. Because of the study’s limitations, though some efficacy for COVID-19 pneumonia, oxygen saturation, and CRP levels in plasma, for instance, has been observed, the reliability and statistical value of the data are modest; only a double-blind, randomized controlled trial in future studies can provide a conclusive response [168].

**Figure 2:**
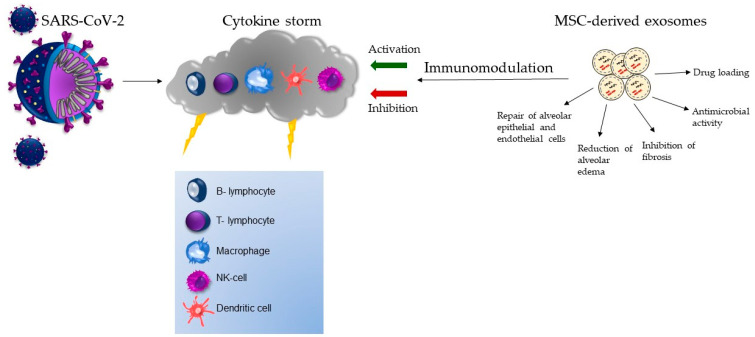
The immunomodulatory potential of MSC-derived exosomes in the management of COVID-19-associated cytokine storm.

It is important to note that while exosomes hold promise as a cell-free therapy for COVID-19, further research and clinical trials are needed to fully understand their effectiveness, safety, and optimal administration routes. Nonetheless, exosomes present an exciting avenue for developing novel therapeutic strategies to combat COVID-19 and other diseases. The action of MSC-derived exosomes in the therapy of COVID-19-associated CS is depicted in Figure 2.

## 7. Conclusions and Future Perspectives

In 2022, Karakaş et al. reported that more than 70 clinical trials have used MSCs in COVID-19 therapy with encouraging results [169]. However, cell-based therapies have multiple disadvantages over cell-free therapies, including tumorigenic potential, cell rejection, unwanted differentiation, immunogenicity, and iatrogenicity. Moreover, cell-free factors are easier to stockpile, are not demanding in terms of storage and transport requirements, and are easier to control quantitatively and qualitatively [170]. Another issue is that cell expansion and the in vitro manipulation of MSCs can cause abnormal function, thus causing non-responses to such therapy. Fortunately, many of these issues have been successfully overcome.

Nevertheless, cell-free therapy is the most promising up-and-coming alternative to cell-based approaches. Apart from the abovementioned limitations, cell-free therapies, e.g., CM, are also advantageous thanks to their high output potential and efficiency, bypassing the need for a high quantity of cells, so they are also beneficial from the economic standpoint. On top of that, CM production can also be tweaked by various manipulations, such as pre-conditioning, to produce a desirable final “tailored” product. Compared to the therapeutic potential of MSCs, cell-free CMs have similar effects in terms of immunomodulatory potential and anti-inflammatory and regenerative properties [141].

Exosomes are also a cell-free approach with a high potential for therapeutic benefit. Apart from their already-discussed capacity for the effective management of the CS, there are other promising avenues of how exosomes can be utilized in infectious disease management, e.g., in terms of vaccine development. As mentioned, in 2007, Kuate et al. studied the original SARS-CoV causing SARS, trying to find an effective vaccine based on exosomes. This exosomal vaccine induced an effective immune response [151]. In 2022, Wang et al. published a study that presented an alternative to established COVID-19 vaccination protocols. The authors manufactured and tested a vaccine that combined the recombinant SARS-CoV-2 receptor-binding domain conjugated to lung-derived exosomes. This inhalable vaccine was stable at room temperature for more than three months and showed promising effects in hamsters, providing another clue of how exosomes can be implemented for COVID-19 or similar disease management [171]. Exosome-based vaccines can be categorized under the umbrella of a modern area with a great future outlook—the research of engineered exosomes—which are defined as exosomes that have been modified either on their surface by adding various molecules to their membrane or by a therapeutic agent introduced internally into the vesicle. Through such modifications, exosomes can be used for targeted drug delivery, circumventing the suboptimal targeting capacity of naturally occurring exosomes [110]. Engineered exosomes can be modified biologically, immunologically, physically, and chemically to optimize their properties best, enabling a superior therapeutic effect [172]. Engineered exosomes, or more broadly, EVs, have also been studied in COVID-19. Scott et al. engineered EVs directed against the SARS-CoV-2 spike protein, neutralizing the virus [173]. Nazerian et al. chose an original and personalized approach. The authors implemented a two-step strategy; the authors used 8P9R chimeric peptide and soluble(s)ACE2 delivered via exosome–liposome hybrids. As the first measure, the 8P9R chimeric peptide inhibited virus replication, preventing the progression to a hyper-inflammatory state. In the second step, the introduction of sACE2 inhibited the activation of inflammatory pathways leading to the CS [174]. Cell-free approaches seem to have several advantages over cell-based therapies, but many downsides still need to be addressed. The main issue is the lack of standardization of CM and exosome preparation protocols.

It is important to continue the research endeavors of finding the most effective therapy for COVID-19. This is true from various perspectives. Firstly, it is very hard to predict the emergence of new variants of the SARS-CoV-2 virus, which could lead to new outbreaks of the disease. Secondly, and most importantly, the further development of COVID-19 therapies will serve as a modeling tool for other pandemics that may strike in the future. Cell-free approaches are at the forefront of research interest around the globe and will continue to refine the state-of-the-art management of countless diseases, from non-communicable diseases to communicable diseases such as COVID-19—or perhaps a future COVID-2x pandemic?

## Figures and Tables

**Table 1:** Selected studies on the therapeutic effect of stem-cell-derived CM on lung injury animal models.

Type of Donor Cell	Animal Model	Induction of Lung Injury	Biological Mechanism	Therapeutic Outcome	Ref.
MSCs	Wild-type male C57BL/6 mice	Lipopolysaccharide-induced lung injury (ALI)	Enhanced expression of Ym1 and decreased expression of inducible nitric oxide synthase	Attenuation of lung inflammation; promotion of wound healing via M2 alveolar macrophage phenotype activation	[62]
MSCs	Wild-type male C57BL/6 mice	Lipopolysaccharide-induced lung injury (ALI)	Enhancement of the neutrophil’s apoptosis and anti-apoptotic molecule (Bcl-xL and Mcl-1) expression reduction; inhibition of the NF-κB pathway	Attenuation of IL-6, macrophage inflammatory protein 2 (MIP-2); reduction in neutrophil accumulation and activity in injured lung tissue	[77]
BM-MSCs	Male C57BL/6 mice	Lipopolysaccharide-induced lung injury (ALI)	Increased expression of miR-214, which activates α-epithelial sodium channel in alveolar type 2 epithelial cells (AT2) and H441 cells	Improved alveolar fluid clearance, thus facilitating edema fluid resolution	[84]
BM-MSCs	Male C57BL/6 mice	Lipopolysaccharide-induced lung injury (ALI)	Increased protein and miR-34c expression of the γ-epithelial sodium channel in AT2 and H441 cells	Increased viability of AT2 and H441 cells; increased clearance of edema fluid; promotion of repair processes	[85]
UC-MSCs	C57BL/6 mice	Lipopolysaccharide-induced lung injury (ALI)	Downregulation of myeloperoxidase activity, IL1β, IL8, and TNFα; increased expression of Arginase-1 and inducible nitric oxide synthase in lung tissue	Attenuation of lung inflammation; promotion of efferocytosis; modulation of anti-inflammatory polarization of lung macrophages	[69]
iPSCs	Wild-type male C57BL/6 mice	Lipopolysaccharide-induced lung injury (ALI)	Promotion of endogenous leukemia inhibitory factor (LIF) in the inhibition of neutrophils’ transendothelial migration	Reduction in histopathological changes (pulmonary endothelium permeability and leakage and neutrophil chemotaxis); attenuation of the severity of ALI	[87]
iPSCs	Wild-type male C57BL/6 mice	Ventilator-induced lung injury (VILI)	Suppression of PI3K/Akt signaling	Decrease in high-tidal-volume-induced VILI-related inflammatory processes and HMGB1 and PAI-1 production	[88]
BM-MSCs	Sprague Dawley neonatal rats	Oxygen-induced lung injury	Enhanced secretion of antioxidant STC-1	Prevention of pulmonary hypertension; preservation of alveolar growth	[66]

**Table 2:** Selected studies on the therapeutic effect of stem-cell-derived EVs on respiratory disease animal models.

Type of Donor Cell Derivate	Animal Model	Induction of Lung Injury	Biological Mechanism	Therapeutic Outcome	Ref.
BM-MSC-derived microvesicles	Wild-type male C57BL/6 mice	*Escherichia coli*-induced pneumonia (ALI)	KGF secretion; enhanced monocyte phagocytosis of bacteria; prestimulation of MSCs with Toll-like receptor 3 agonists; decreased TNF secretion and increased IL-10 secretion	Increased survival rate; decreased lung inflammation and protein permeability; elimination of bacteria	[111]
MSC-derived EVs	C57BL/6 mice	Lipopolysaccharide-induced lung injury (ALI)	High expression of miR-27a-3p	Alleviation of ALI; promoting M2 macrophage polarization	[112]
MSC-derived microvesicles	Wild-type male C57BL/6 mice	Lipopolysaccharide-induced lung injury (ALI)	Overexpression of angiopoietin-1 mRNA; decreased TNF secretion and increased IL-10 secretion	A decreased influx of inflammatory cells in injured alveoli; restoration of pulmonary capillaries permeability; attenuation of histological injury	[130]
BM-MSC-derived EVs	Large White–Duroc crossbred pigs	Influenza-virus-induced lung injury (ALI)	Transfer of RNAs from EVs to alveolar cells; increased secretion of IL-10	Inhibition of virus replication, virus-induced apoptosis, and secretion of proinflammatory cytokines	[136]
MSC-derived exosomes	C57BL/6 mice	Lipopolysaccharide-induced lung injury (ALI)	Transfer of stem-cell-derived mitochondria components to alveolar macrophages	Restoration of mitochondrial integrity; shift of alveolar macrophages to anti-inflammatory phenotype; mitigating lung inflammation	[137]
BM-MSC-derived exosomes	Male ICR mice	Sulfur-mustard-induced lung injury (ALI)	Upregulation of G protein-coupled receptor family C group 5 type A; facilitation of the expression and relocalization of junction proteins	Protection against pulmonary edema; inhibition of alveolar cell apoptosis; recovery of epithelial barrier	[133]

**Table 3:** Therapeutic effects of CM in COVID-19.

Type of Reaction	Therapeutic Effect
Anti-inflammatory	reduction in excessive immune responses reduction in inflammation prevention of CSs
Immunomodulatory	regulation leading to a balanced immune response affecting T cells, B cells, and NK cells
Regenerative	promotion of tissue regeneration and repair stimulation of proliferation and differentiation of endogenous progenitor cells
Anti-viral	inhibition of viral replication via direct anti-viral molecules/properties enhancement of anti-viral defense mechanisms

## Data Availability

Not applicable.
